# Supplementary material for: Study on the enhancement of low carbon-to-nitrogen ratio urban wastewater pollutant removal efficiency by adding sulfur electron acceptors
Source: PLoS One. 2024 Oct 24;19(10):e0310222. doi: 10.1371/journal.pone.0310222 (PMC11500869; doi:10.1371/journal.pone.0310222)
Supplement: Attachment — Submitted filename: Response to Reviewers.docx [file pone.0310222.s001.docx]

**Response to Reviewers**

Dear Editors and Reviewers,

The enclosed manuscript entitled “Study on the Enhancement of Low Carbon-to-Nitrogen Ratio Urban Wastewater Pollutant Removal Efficiency by Adding Sulfur Electron Acceptors” is a revised submission (Manuscript Number: PONE-D-24-01884). Many thanks to the editor and reviewers for their valuable comments on the paper. So we have made substantial changes to it, including the language and writing, the introduction, and logical description. All the authors wish it could be considered for publication in *PLOS ONE*.

The comments from the editor/reviewers and the response to editor/reviewers are described as follows,

**Journal Requirements:**

**1.When submitting your revision, we need you to address these additional requirements.**

**Reply:** Additional requirements has been addressed rightly.

**2＆3.We note that the grant information you provided in the ‘Funding Information’ and ‘Financial Disclosure’ sections do not match. funding information should not appear in the Acknowledgments section or other areas of your manuscript**

**Reply:**Funding Information has been removed from the manuscript and update in cover letter.

**4.We note that your Data Availability Statement is currently as follows: [All relevant data are within the manuscript and its Supporting Information files]**

**Reply:**Yes , I confirmed that my submission contains all raw data required to replicate the results of your study.

**Reviewer #1:**

**1) The abstract part seems shallow; not addressed the main points of the study.**

**Reply:**

→ ‘We had modified the Abstract to highlight the purpose of this article, the experimental approach, the results and future research..’

**2) In introduction part, you provided general issues about C/N. its better if you concentrated on the reality of your study areas. The introduction part also required to summarize more than this.**

**Reply:**

→ The reality of your study areas has been Added into the introduction and the logical description in introduction had been modified .

**3) on line 138, you stated figure 1. But there wasn’t it**

**Reply:**

→ Figure 1 had been shown in the bottom of Submission.

**4) Your research design and methodology the way of sample collection and preservation needs modification**

**Reply:**

**→** The design and methodology the way of sample collection and preservation has been revised in the manuscript.

## **‘**2.2 Inoculation sludge and synthetic sewage

In this study, the inoculated sludge came from a sewage treatment plant in Nansha, Guangzhou, which operated stably for a long time. Before the sludge was inoculated, the sludge was cleaned to remove impurities in the sludge. 5L of anaerobic sludge was inoculated in each of the two UASB bioreactors with a sludge concentration of about 4000 to 5500 mg / L. At the same time, 3L aerobic sludge was inoculated in the aerobic reactor and 30% polyurethane fillers was added to the aerobic sludge for mixing.

In order to investigated the purification capacity of bioreactor to low C / N urban sewage, Synthetic low C/N ratio wastewater prepared with tap water was used in this experiment. Ingredients included C_6_H_12_O_6_(carbon source, 110 mg COD/L); NH_4_Cl(source of NH_3_-N,25mg N/L); NaH_2_PO_4_(source of P, 1.15mg P/L). At the same time, 1.0ml/L trace elements concentrate was added to the domestic wastewater[24]. The trace element concentrate is composed of :EDTA 5000 mg/L，FeCl _2_  3000 mg/L，MnCl_2_·4H_2_O 1000mg/L, ZnCl_2_·7H_2_O 450 mg/L, CuCl_2_ ·5H_2_O 270 mg/L， NiCl _2_ ·6H _2_ O 200 mg/L ， CoCl _2_ ·6H _2_ O 240 mg/L.

## 2.3 Chemical analysis

All samples of the water were filtered through 0.45μm filter paper before analysis. COD concentration using COD rapid measurement analyzer (DR1010,HACH, USA). Ammonia nitrogen (NH_4_^+^-N) was measured by ammonia nitrogen tester (5B-3N, LianHua, China). The nitrite nitrogen (NO_2_^-^-N), nitro nitrogen (NO_3_^-^-N), total , Sulfate (SO_4_^2-^-S) and the sulfide (S^2-^-S) was measured using a multi-parameter analyzer (DR3900, HACH, USA). The WTW real-time water quality testing equipment (Multi 3420, WTW, Germany) was used to monitor the pH and temperature.All water samples were measured in triplicate.**’**

**5）the style you wrote the equations not followed the standard**

**Reply:**

**→** The equations has been revised in the manuscript. For example

$1/24 C_{6}H_{12}O_{6}+1/4 O_{2}\to1/4 \mathrm{CO}_{2}+1/4 H_{2}O$

ΔG= -120.1kJ/mol

**6）your result description wasn’t smart. It needs modification**

**Reply:**

**→** The logical description and structure of **result** has been revised in the manuscript.

**7）would you followed the reference writing style of PLOSE ONE?**

**Reply:**

**→** The reference writing style has been revised in the manuscript

1. Song H, Feng J, Zhang L, et al.Advanced treatment of low C/N ratio wastewater treatment plant effluent using a denitrification biological filter: Insight into the effect of medium particle size and hydraulic retention time.Environmental Technology & Innovation, 2021, 24: 102044.[https://doi.org/10.1016/j.eti.2021.102044](https://doi.org/10.1016/j.eti.2021.102044)
2. Preisner M, Neverova-Dziopak E, Kowalewski Z. Analysis of eutrophication potential of municipal wastewater. Water Science and Technology, 2020, 81(9): 1994-2003.https://doi.org/10.2166/wst.2020.254
3. Li D, Li W, Zhang D, et al. Performance and mechanism of modified biological nutrient removal process in treating low carbon-to-nitrogen ratio wastewater. Bioresource Technology, 2023, 367: 128254. <https://doi.org/10.1016/j.biortech.2022.128254>
4. Yuan C, Zhao F, Zhao X, et al. Woodchips as sustained-release carbon source to enhance the nitrogen transformation of low C/N wastewater in a baffle subsurface flow constructed wetland. Chemical Engineering Journal, 2020, 392: 124840.https://doi.org/10.1016/j.cej.2020.124840
5. Huang W, Gong B, Wang Y, et al.Metagenomic analysis reveals enhanced nutrients removal from low C/N municipal wastewater in a pilot-scale modified AAO system coupling electrolysis.Water Research, 2020, 173: 115530.https://doi.org/10.1016/j.watres.2020.115530
6. Awasthi M K, Sarsaiya S, Wainaina S, et al. Techno-economics and life-cycle assessment of biological and thermochemical treatment of bio-waste. Renewable and Sustainable Energy Reviews, 2021, 144: 110837. [https://doi.org/10.1016/j.rser.2021.110837](https://doi.org/10.1016/j.rser.2021.110837)
7. Duan S, Zhang Y, Zheng S. Heterotrophic nitrifying bacteria in wastewater biological nitrogen removal systems: A review.Critical Reviews in Environmental Science and Technology, 2022, 52(13): 2302-2338. <https://doi.org/10.1080/10643389.2021.1877976>
8. Wang B, Hu H, Huang S, et al.Simultaneous nitrate and sulfate biotransformation driven by different substrates: comparison of carbon sources and metabolic pathways at different C/N ratios.RSC Advances, 2023, 13(28): 19265-19275.
9. Wu L, Wei W, Xu J, et al.Denitrifying biofilm processes for wastewater treatment: developments and perspectives.Environmental Science: Water Research & Technology, 2021, 7(1): 40-67.https://doi.org/10.1039/D3RA02749J
10. Hellinga C, Schellen A, Mulder J W, et al. The SHARON process: an innovative method for nitrogen removal from ammonium-rich waste water. Water science and technology, 1998, 37(9): 135-142.
11. Jin R C, Yang G F, Yu J J, et al. The inhibition of the Anammox process: a review. Chemical engineering journal, 2012, 197: 67-79.
12. Lotti T, Kleerebezem R, van Erp Taalman Kip C, et al. Anammox growth on pretreated municipal wastewater. Environmental science & technology, 2014, 48(14): 7874-7880.
13. Van Dongen U, Jetten M S M, van Loosdrecht M C M. The SHARON®-Anammox® process for treatment of ammonium rich wastewater. Water science and technology, 2001, 44(1): 153-160.
14. Sousa A F S. The SHARON-anammox process for the treatment of ammonium-rich liquid residues produced by the anaerobic digestion of municipal solid wastes: a preliminary evaluation. Institute of Environmental Geology and Geoengineering, National Research Council, 2016.
15. Soliman M, Eldyasti A. Ammonia-Oxidizing Bacteria (AOB): opportunities and applications—a review. Reviews in Environmental Science and Bio/Technology, 2018, 17(2): 285-321.
16. Lotti T, Kleerebezem R, Lubello C, et al. Physiological and kinetic characterization of a suspended cell anammox culture. Water research, 2014, 60: 1-14.
17. Kumar U, Panneerselvam P, Gupta V V S R, et al. Diversity of sulfur-oxidizing and sulfur-reducing microbes in diverse ecosystems. Advances in Soil Microbiology: Recent Trends and Future Prospects: Volume 1: Soil-Microbe Interaction, 2018: 65-89.https://doi.org/10.1007/978-981-10-6178-3_4
18. Zhang L, Zhang S, Wang S, et al. Enhanced biological nutrient removal in a simultaneous fermentation, denitrification and phosphate removal reactor using primary sludge as internal carbon source. Chemosphere, 2013, 91(5): 635-640.
19. Nguyen T A, Juang R S. Treatment of waters and wastewaters containing sulfur dyes: A review. Chemical engineering journal, 2013, 219: 109-117.
20. Brown K A. Sulphur in the environment: a review. Environmental Pollution Series B, Chemical and Physical, 1982, 3(1): 47-80.
21. Selvasembian R, Gwenzi W, Chaukura N, et al. Recent advances in the polyurethane-based adsorbents for the decontamination of hazardous wastewater pollutants. Journal of Hazardous Materials, 2021, 417: 125960. [https://doi.org/10.1016/j.jhazmat.2021.125960](https://doi.org/10.1016/j.jhazmat.2021.125960)
22. Shao M F, Zhang T, Fang H H P. Sulfur-driven autotrophic denitrification: diversity, biochemistry, and engineering applications Applied microbiology and biotechnology, 2010, 88: 1027-1042.https://doi.org/10.1007/s00253-010-2847-1
23. Huang S, Yu D, Chen G, et al. Realization of nitrite accumulation in a sulfide-driven autotrophic denitrification process: Simultaneous nitrate and sulfur removal. Chemosphere, 2021, 278: 130413. <https://doi.org/10.1016/j.chemosphere.2021.130413>
24. Zhang Y, Zhang L, Li L, et al.A novel elemental sulfur reduction and sulfide oxidation integrated process for wastewater treatment and sulfur recycling.Chemical Engineering Journal, 2018, 342: 438-445. <https://doi.org/10.1016/j.cej.2018.02.105>
25. Li D, Li W, Zhang D, et al. Performance and mechanism of modified biological nutrient removal process in treating low carbon-to-nitrogen ratio wastewater. Bioresource Technology, 2023, 367: 128254. <https://doi.org/10.1016/j.biortech.2022.128254>
26. Wang X, Jiang C, Wang D, et al.Quorum sensing responses of activated sludge to free nitrous acid: Zoogloea deformation, AHL redistribution, and microbiota acclimatization.Water Research, 2023, 238: 119993. <https://doi.org/10.1016/j.watres.2023.119993>
27. Xu Z, Dai X, Chai X.Effect of different carbon sources on denitrification performance, microbial community structure and denitrification genes.Science of the Total Environment, 2018, 634: 195-204. <https://doi.org/10.1016/j.scitotenv.2018.03.348>
28. Wu D, Ekama G A, Chui H K, et al.Large-scale demonstration of the sulfate reduction autotrophic denitrification nitrification integrated (SANI®) process in saline sewage treatment.Water research, 2016, 100: 496-507. <https://doi.org/10.1016/j.watres.2016.05.052>
29. Liamleam W, Annachhatre A P.Electron donors for biological sulfate reduction.Biotechnology advances, 2007, 25(5): 452-463. <https://doi.org/10.1016/j.biotechadv.2007.05.002>
30. Sharp R, Khunjar W, Daly D, et al. Nitrogen removal from water resource recovery facilities using partial nitrification, denitratation-anaerobic ammonia oxidation (PANDA). Science of The Total Environment, 2020, 724: 138283. <https://doi.org/10.1016/j.scitotenv.2020.138283>
31. He Q, Cheng Z, Zhang D, et al.A sulfur-based cyclic denitrification filter for marine recirculating aquaculture systems.Bioresource Technology, 2020, 310: 123465.https://doi.org/10.1016/j.biortech.2020.123465
32. Wang H, Jiang C, Wang X, et al.Application of internal carbon source from sewage sludge: A vital measure to improve nitrogen removal efficiency of low C/N wastewater.Water, 2021, 13(17): 2338.https://doi.org/10.3390/w13172338
33. Di Capua F, Pirozzi F, Lens P N L, et al.Electron donors for autotrophic denitrification.Chemical Engineering Journal, 2019, 362: 922-937. <https://doi.org/10.1016/j.cej.2019.01.069>
34. Fu X, Hou R, Yang P, et al. Application of external carbon source in heterotrophic denitrification of domestic sewage: A review. Science of The Total Environment, 2022, 817: 153061.https://doi.org/10.1016/j.scitotenv.2022.153061
35. Liu F, Wang S, Zhang X, et al.Nitrate removal from actual wastewater by coupling sulfur-based autotrophic and heterotrophic denitrification under different influent concentrations.Water, 2021, 13(20): 2913.https://doi.org/10.3390/w13202913
36. Cui Y X, Biswal B K, Guo G, et al.Biological nitrogen removal from wastewater using sulphur-driven autotrophic denitrification.Applied microbiology and biotechnology, 2019, 103: 6023-6039.https://doi.org/10.1007/s00253-019-09935-4
37. Fan C, Zhou W, He S, et al.Sulfur transformation in sulfur autotrophic denitrification using thiosulfate as electron donor.Environmental Pollution, 2021, 268: 115708.https://doi.org/10.1016/j.envpol.2020.115708
38. Vázquez-Fernández A, Suárez-Ojeda M E, Carrera J. Review about bioproduction of Volatile Fatty Acids from wastes and wastewaters: Influence of operating conditions and organic composition of the substrate. Journal of Environmental Chemical Engineering, 2022, 10(3): 107917. <https://doi.org/10.1016/j.jece.2022.107917>
39. Wang D, Li Y, Cope H A, et al. Intracellular polyphosphate length characterization in polyphosphate accumulating microorganisms (PAOs): Implications in PAO phenotypic diversity and enhanced biological phosphorus removal performance. Water Research, 2021, 206: 117726. <https://doi.org/10.1016/j.watres.2021.117726>
40. Tayà C, Guerrero J, Suárez-Ojeda M E, et al. Assessment of crude glycerol for Enhanced Biological Phosphorus Removal: Stability and role of long chain fatty acids. Chemosphere, 2015, 141: 50-56.https://doi.org/10.1016/j.chemosphere.2015.05.067
41. Brahmacharimayum B, Mohanty M P, Ghosh P K.Theoretical and practical aspects of biological sulfate reduction: a review.Global NEST Journal, 2019, 21(2): 222-244.https://doi.org/10.30955/gnj.002577
42. Saad S A, Welles L, Lopez-Vazquez C M, et al.Sulfide effects on the anaerobic kinetics of phosphorus-accumulating organisms.of the World Congress on Anaerobic Digestion, Santiago de Compostela, Spain.2013.
43. Ikumi D S, Ekama G A.Plantwide modelling–anaerobic digestion of waste sludge from parent nutrient (N and P) removal systems.Water SA, 2019, 45(3): 305-316.

https://doi.org/[10.17159/wsa/2019.v45.i3.6698](https://doi.org/10.17159/wsa/2019.v45.i3.6698)

44. Lu X, Duan H, Oehmen A, et al.Achieving combined biological short-cut nitrogen and phosphorus removal in a one sludge system with side-stream sludge treatment.Water Research, 2021, 203: 117563. <https://doi.org/10.1016/j.watres.2021.117563>
45. Dahl C.A biochemical view on the biological sulfur cycle.Environmental Technologies to Treat Sulfur Pollution: Principles and Engineering, 2020, 2: 55-96.

<https://doi.org/10.2166/9781789060966_0055>

46. Gunarathne V, Rajapaksha A U, Vithanage M, et al.Hydrometallurgical processes for heavy metals recovery from industrial sludges.Critical Reviews in Environmental Science and Technology, 2022, 52(6): 1022-1062. <https://doi.org/10.1080/10643389.2020.1847949>
47. Chatla A, Almanassra I W, Abushawish A, et al.Sulphate removal from aqueous solutions: State-of-the-art technologies and future research trends.Desalination, 2023: 116615.https://doi.org/10.1016/j.desal.2023.116615
48. Plugge C M, Zhang W, Scholten J C M, et al. Metabolic flexibility of sulfate-reducing bacteria. Frontiers in microbiology, 2011, 2: 81.https://doi.org/10.3389/fmicb.2011.00081
49. Aüllo T, Ranchou-Peyruse A, Ollivier B, et al.Desulfotomaculum spp.and related gram-positive sulfate-reducing bacteria in deep subsurface environments.Frontiers in microbiology, 2013, 4: 362.https://doi.org/10.3389/fmicb.2013.00362
50. Qiu Y Y, Guo J H, Zhang L, et al.A high-rate sulfidogenic process based on elemental sulfur reduction: Cost-effectiveness evaluation and microbial community analysis.Biochemical engineering journal, 2017, 128: 26-32. <https://doi.org/10.1016/j.bej.2017.09.001>
51. Zhong W H, Zhu B T, Zhao C G, et al.Factors affecting nitrogen removal from aquaculture wastewater by Rhodobacter azotoformans YLK20.Microbiol China, 2019, 46(9): 2146-2156.https://doi.org/[10.13344/j.microbiol.china.190291](http://dx.doi.org/10.13344/j.microbiol.china.190291)
52. Chandran V, Shaji H, Mathew L.Endophytic microbial influence on plant stress responses.Microbial endophytes.Woodhead Publishing, 2020: 161-193. <https://doi.org/10.1016/B978-0-12-819654-0.00007-7>
53. JIANG Peng, ZHAO Chun-Gui, JIA Ya-Qiong, YANG Su-Ping.Effects of nitrite on ammonia-nitrogen removal and nitrite-nitrogen as well as photopigment biosynthesis of Marichromatium gracile YL28.Microbiology China, 2015, 42(7): 1216-1223
54. Green S J, Prakash O, Jasrotia P, et al. Denitrifying bacteria from the genus Rhodanobacter dominate bacterial communities in the highly contaminated subsurface of a nuclear legacy waste site. Applied and environmental microbiology, 2012, 78(4): 1039-1047. <https://doi.org/10.1128/AEM.06435-11>
55. Wang X, Jiang C, Wang D, et al.Quorum sensing responses of activated sludge to free nitrous acid: Zoogloea deformation, AHL redistribution, and microbiota acclimatization.Water Research, 2023, 238: 119993. <https://doi.org/10.1016/j.watres.2023.119993>
56. An W, Guo F, Song Y, et al.Comparative genomics analyses on EPS biosynthesis genes required for floc formation of Zoogloea resiniphila and other activated sludge bacteria.Water Research, 2016, 102: 494-504. <https://doi.org/10.1016/j.watres.2016.06.058>
57. Zhao C, Zhang L Y, Ma T, et al.The synergistic effect for C/N removal by UASB reactor on mature landfill leachate.Research of Environmental Sciences, 2019, 31(11): 1913-1920.
58. Kadnikov V V, Mardanov A V, Beletsky A V, et al. Genome of the candidate phylum Aminicenantes bacterium from a deep subsurface thermal aquifer revealed its fermentative saccharolytic lifestyle. Extremophiles, 2019, 23: 189-200. https://doi.org/10.1007/s00792-018-01073-5
59. McIlroy S J, Starnawska A, Starnawski P, et al.Identification of active denitrifiers in full‐scale nutrient removal wastewater treatment systems.Environmental microbiology, 2016, 18(1): 50-64. https://doi.org/10.1111/1462-2920.12614
60. Zhang M, Wang X, Zhang D, et al.Food waste hydrolysate as a carbon source to improve nitrogen removal performance of high ammonium and high salt wastewater in a sequencing batch reactor.Bioresource Technology, 2022, 349: 126855.https://doi.org/10.1016/j.biortech.2022.126855
61. Dabert P, Sialve B, Delgenès J P, et al.Characterisation of the microbial 16S rDNA diversity of an aerobic phosphorus-removal ecosystem and monitoring of its transition to nitrate respiration.Applied microbiology and biotechnology, 2001, 55: 500-509.https://doi.org/10.1007/s002530000529
62. Qiu Y Y, Zou J, Xia J, et al. Adaptability of sulfur-disproportionating bacteria for mine water remediation under the pressures of heavy metal ions and high sulfate content. Water Research, 2024, 249: 120898.https://doi.org/10.1016/j.watres.2023.120898
63. Xu Y N, Chen Y. Advances in heavy metal removal by sulfate-reducing bacteria. Water Science and Technology, 2020, 81(9): 1797-1827.https://doi.org/10.2166/wst.2020.227

Ouyang J, Miao Q, Wei D, et al. Removal of Cr (VI) and microbial community analysis in PCB wastewater treatment based on the BESI® process. Plos one, 2023, 18(8): e0290023.https://doi.org/10.1371/journal.pone.029002

**Reviewer #2:**

**1） The figures in this manuscript seems very blur**.

**Reply:**

**→** The figures in the manuscript has been revised .

**2＆3）What is the organic matter used for the synthetic wastewater preparation?**

**What is the “The necessary trace elements” added to the domestic wastewater?**

**Reply:**

**→** The organic matter used for the synthetic wastewater preparation and the description of wastewater preparation has been modified clearly.

“In order to investigated the purification capacity of bioreactor to low C / N urban sewage, Synthetic low C/N ratio wastewater prepared with tap water was used in this experiment. Ingredients included C_6_H_12_O_6_(carbon source, 110 mg COD/L); NH_4_Cl(source of NH_3_-N,25mg N/L); NaH_2_PO_4_(source of P, 1.15mg P/L). At the same time, 1.0ml/L trace elements concentrate was added to the domestic wastewater[24]. The trace element concentrate is composed of :EDTA 5000 mg/L，FeCl _2_  3000 mg/L，MnCl_2_·4H_2_O 1000mg/L, ZnCl_2_·7H_2_O 450 mg/L, CuCl_2_ ·5H_2_O 270 mg/L， NiCl _2_ ·6H _2_ O 200 mg/L ， CoCl _2_ ·6H _2_ O 240 mg/L.”

**4）In my opinion, it is better to add a table to show the operational parameters for phase I and II.**

**Reply:**

**→** The operational parameters for phase I and II was shown blow.

**Table 1 The experimental parameters of phases I and II**

| **Parameter** | **Phase Ⅰ (Day 1–30)** | **Phase ⅠⅠ (Day 31-90)** |
| --- | --- | --- |
| **C:N ratio** | 4.4 | 4.4 |
| **SRT(h)** | 12 | 12 |
| **NH4 ^+^（mg/L）** | 25 | 25 |
| **SO_4_^2−^ （mg/L）** | 0 | 100 |
| **HRT** | 20 | 20 |

**5） What is the meaning of “dynamic changes”?**

**Reply:**

**→**Dynamic changes refers to change over time, in the environment or other factors, and is often used to describe the continuous adjustment and adaptation of systems, processes or behaviours.But this word sound like an Chinglish rather than academic words,so I replace this word into more academic wold in detail like “the COD concentration changes of each reactor”.

**6） The writing English should be improved**

**Reply:**

**→**The manuscript has been modified for word spelling, sentence tense, and logical structure.

**7）Why did the authors add polyurethane fillers to the aerobic reactor?**

**Reply:**

**→**The main functions of polyurethane fillers include improving the efficiency and stability of biochemical systems. They achieve this through simple physical adsorption and the attraction of positive and negative charges, firmly fixing microorganisms onto the carrier. This effectively reduces ammonia nitrogen and total nitrogen in wastewater. Additionally, they possess the ability to cut bubbles and optimize space utilization, while effectively inhibiting the toxic effects of refractory substances and toxic substances on microbial communities, and significantly reducing the production of residual sludge. Their advantage lies in the design combining macropores and micropores: macropores maintain good contact conditions for gas, liquid, and solid phases, while micropores are used to immobilize microorganisms. The hydrophilic active groups can form ionic or covalent bonds with the active groups in microbial peptide chains, thereby immobilizing microorganisms and bioenzymes on the carrier. This design not only increases the load capacity of microorganisms but also enhances the system's tolerance to load, toxic inhibitory substances, pH, and temperature fluctuations.The description of polyurethane fillers in manuscript has been uploaded.

Many thanks to the editor and reviewer for their valuable comments and suggestions. We have also tried our best to make much revisions on the manuscript. We sincerely hope that it can be published in the journal of PLOS ONE.

Sincerely Yours,

Li Wei
